# Reply: Is thymidylate synthase a reliable predictor for response and survival during hepatic arterial infusion for hepatic metastasis from colorectal cancer

**DOI:** 10.1038/sj.bjc.6603426

**Published:** 2006-10-17

**Authors:** E Goekkurt, J Stoehlmacher

**Affiliations:** 1 Department of Internal Medicine Haematology and Medical Oncology, Fetschestre I, University Hospital Carl Gustav Carus, University Dresden, 74 Dresden, Dresden 1307, Germany

**Sir**,

We appreciate the opportunity to give a comment on the interesting letter by Ferretti *et al* in this issue. The need of a comprehensive combined analysis of multiple germline polymorphisms in genes involved in the folate metabolism and expression of these genes and their proteins in view of corresponding expression data is demanded by the authors for prediction of efficacy and toxicity of 5-fluorouracil (5-FU)-based chemotherapy. We agree to this statement. Our group conducted a combined analysis of polymorphisms of TS and MTHFR (folate pathway) as well as polymorphisms of GSTP1, ERCC1 and ERCC2, enzymes with potential impact on cisplatin efficacy (Goekkurt *et al*, 2006). The finding that our combined analyses of TS 5′ and GSTP1 genotypes resulted in a significant association with response and overall survival in our patient population (advanced gastric cancer patients treated with 5-FU/cisplatin as first-line palliative treatment) encourages for combined and more comprehensive analyses of more than one polymorphism in more than one metabolic or regulatory pathway. There are examples in the literature already for comprehensive genotype analyses for gastric cancer and also for colorectal cancer (Stoehlmacher *et al*, 2004; Ruzzo *et al*, 2006). We are aware of the fact that our study is limited to few genotypes, and, as mentioned in our discussion, more genetic factors (polymorphisms) should be considered for the explanation of inter-individual variability in chemotherapy response, and more so in case of application of combination chemotherapy. Therefore, we mentioned our ongoing prospective study in a large phase III trial in advanced gastric cancer including a panel of at least 26 polymorphisms in genes of platinum and folate metabolism as well as in DNA-repair genes. However, performing such comprehensive analyses must take into account the relevance of certain polymorphisms for the clinical setting, for example very low frequencies of variant genotypes (e.g. dihydropyrimidine dehydrogenase (DPD) polymorphism). Furthermore, a functional impact of a certain polymorphism on gene/protein expression or protein function should be evaluated as clear as possible prior to entering a translational study (e.g. unclear functionality of orotate phosphoribosyl transferase (OPRT) or uridine monophosphate kinase (UMPK) polymorphisms).

In contrast to gene/protein expression data, which always display a cross-section of a dynamic process, genotypes remain stable and can be determined easily either from normal as well as from tumour tissue. As tumour tissue is not always available, especially in the advanced tumour setting, genotyping of normal host cells (e.g. leucocytes) remain easily accessible and cost-effective at any time of treatment. Despite, an existing controversially discussion about the value of host *vs* tumour genotyping for cancer pharmacogenetic studies, the use of germline DNA is validated with a minor source of error due to little discrepancies between the host and tumour genome (Marsh *et al*, 2005). The use of expression data, if available, may be very helpful for pharmacogenomic studies in cancer. However, expression data do not provide information about alteration of protein function, for example, due to germline polymorphisms or tumour-specific mutations. Of course if applicable, tumour-specific mutations should be taken into account in pharmacogenetic analyses as it is the case in non-small-cell lung cancer and in the use of inhibitors of EGFR.

In conclusion, we agree with the demand for a comprehensive combined analysis in cancer pharmacogenetic studies including several relevant and reliable factors. So far, the process of identifying the most important factors is still ongoing and reveals controversial results. Maybe even more important, there are currently no sufficient statistical models to incorporate the different impact of frequency and functionality of a single polymorphism for the prediction value of the whole analysis. For most polymorphisms, its functional significance is not even clear. Especially before that background, we have to incorporate as much information in our pharmacogenetic analyses as possible and perform true comprehensive studies.

If we focus on TS polymorphisms, we note that there are several divergent observations being published so far. In a study by Jakobsen *et al* (2005) in advanced colorectal cancer, for example, TS 5′-UTR 3R/3R genotype carriers showed better clinical outcomes than patients with TS 5′-UTR 2R/2R genotypes. These findings are not in agreement with the hypothesis that the 3R allele is correlated with higher TS mRNA levels and worse clinical outcome. However, the authors did not include the G/C SNP located within the 3R allele which might be an explanation for their opposite findings. Our data in contrast are in line with several observations by other investigators. Recently, in a prospective setting with a large patient cohort (*n*=175), Ruzzo *et al* (2006) could demonstrate the same impact of TS 5′-UTR and GSTP1 polymorphisms than we did on clinical outcome in advanced gastric cancer patients. At least for few genetic markers, such as TS polymorphisms, a lot of data with respect to cancer pharmacogenetic studies exist and prospective validation and evaluation of their potential as stratification parameter is ongoing.

The TS example shows that we will constantly learn in pharmacogenetics and that it needs large and comprehensive studies to find the ‘truth’ in order to develop robust tests for the clinical setting.
